# Population impact and effectiveness of sequential 13-valent pneumococcal conjugate and monovalent rotavirus vaccine introduction on infant mortality: prospective birth cohort studies from Malawi

**DOI:** 10.1136/bmjgh-2020-002669

**Published:** 2020-09-09

**Authors:** Carina King, Naor Bar-Zeev, Tambosi Phiri, James Beard, Hazzie Mvula, Amelia Crampin, Ellen Heinsbroek, Dan Hungerford, Sonia Lewycka, Jennifer Verani, Cynthia Whitney, Anthony Costello, Charles Mwansambo, Nigel Cunliffe, Rob Heyderman, Neil French, VacSurv Consortium, Osamu Nakagomi

**Affiliations:** 1 Department of Global Public Health, Karolinska Institutet, Stockholm, Sweden; 2 Institute for Global Health, University College London, London, London, UK; 3 International Vaccine Access Center, Department of International Health, Johns Hopkins University Bloomberg School of Public Health, Baltimore, Maryland, USA; 4 Centre for Global Vaccine Research, Institute of Infection & Global Health, University of Liverpool, Liverpool, Merseyside, UK; 5 Malawi-Liverpool-Wellcome Trust Clinical Research Programme, College of Medicine, University of Malawi, Blantyre, Malawi; 6 MaiMwana Project, Parent and Child Health Initiative, Lilongwe, Malawi; 7 Karonga Prevention Study, Malawi Epidemiology and Intervention Research Unit, Chilumba, Malawi; 8 Institute of Health & Wellbeing, University of Glasgow, Glasgow, UK; 9 Department of Population Health, London School of Hygiene and Tropical Medicine, London, UK; 10 Nuffield Department of Medicine, Centre for Tropical Medicine, University of Oxford, Oxford, Oxfordshire, UK; 11 Centers for Disease Control and Prevention, Atlanta, Georgia, USA; 12 Hubert Department of Global Health, Rollins School of Public Health, Emory University, Atlanta, Georgia, USA; 13 Ministry of Health, Lilongwe, Malawi; 14 NIHR Global Health Research Unit on Mucosal Pathogens, Division of Infection & Immunity, University College London, London, UK

**Keywords:** pneumococcal disease, vaccines, child health

## Abstract

**Background:**

Pneumococcal conjugate vaccine (PCV) and rotavirus vaccine (RV) are key tools for reducing common causes of infant mortality. However, measurement of population-level mortality impact is lacking from sub-Saharan Africa. We evaluated mortality impact and vaccine effectiveness (VE) of PCV13 introduced in November 2011, with subsequent RV1 roll-out in October 2012, in Malawi.

**Methods:**

We conducted two independent community-based birth cohort studies. Study 1, in northern Malawi (40000population), evaluated population impact using change-point analysis and negative-binomial regression of non-traumatic 14–51-week infant mortality preintroduction (1 January 2004 to 31 September 2011) and postintroduction (1 October 2011 to 1 July 2019), and against three-dose coverage. Study 2, in central Malawi (465 000 population), was recruited from 24 November 2011 to 1 June 2015. In the absence of preintroduction data, individual three-dose versus zero-dose VE was estimated using individual-level Cox survival models. In both cohorts, infants were followed with household visits to ascertain vaccination, socioeconomic and survival status. Verbal autopsies were conducted for deaths.

**Results:**

Study 1 included 20 291 live births and 216 infant deaths. Mortality decreased by 28.6% (95% CI: 15.3 to 39.8) post-PCV13 introduction. A change point was identified in November 2012. Study 2 registered 50 731 live births, with 454 deaths. Infant mortality decreased from 17 to 10/1000 live births during the study period. Adjusted VE was 44.6% overall (95% CI: 23.0 to 59.1) and 48.3% (95% CI: −5.9 to 74.1) against combined acute respiratory infection, meningitis and sepsis-associated mortality.

**Conclusion:**

These data provide population-level evidence of infant mortality reduction following sequential PCV13 and RV1 introduction into an established immunisation programme in Malawi. These data support increasing coverage of vaccine programmes in high-burden settings.

**Key questions**

**What is already known?**

- Several studies have shown post-pneumococcal conjugate vaccine (PCV) introduction reductions in pneumonia cases and hospitalisation in sub-Saharan Africa, and diarrhoea-specific mortality reductions have been attributed to rotavirus vaccine (RV).
- Two studies from South America have reported on PCV effectiveness against mortality, and a retrospective time series analysis from Morocco reported a 30% reduction in acute respiratory infant deaths following PCV introduction. Other estimates of mortality effectiveness and cost-effectiveness have been modelled.
- As yet, no studies have reported prospective empirically observed PCV effectiveness and impact against population-level mortality in sub-Saharan Africa, and none have accounted for RV introduction.

**What are the new findings?**

- We report on the effectiveness and impact of 13-valent PCV, with subsequent RV introduction, on population infant mortality in Malawi.
- We demonstrate significant reductions in all non-traumatic infant mortality among three-dose age-eligible infants following PCV13 introduction in Malawi, in two different locations.
- The reductions in mortality were associated with vaccine coverage. However, we were unable to fully disentangle the additional role of subsequent monovalent RV into the routine extended programme of immunisation (EPI) schedule.

**Key questions**

**What do the new findings imply?**

- PCV13 introduction, as part of a well-established EPI programme in a low-income, high-burden setting alongside RV introduction, is associated with significant reductions in infant mortality.
- This strengthens the growing evidence that investment in PCV and RV scale-up to prevent infant deaths should be prioritised.

## Introduction

Pneumonia remains the leading cause of infectious mortality in children under 5 years old globally. Pneumococcal pneumonia, alongside meningitis and bacteraemia caused an estimated 500 000 annual deaths in 2000, prior to widespread pneumococcal conjugate vaccine (PCV) introduction, with the majority occurring in sub-Saharan Africa.^1 2^ By 2015 this estimate had halved with approximately 300 000 paediatric pneumococcal deaths.^3^ Rotavirus is the leading cause of severe diarrhoea and a significant contributor to infant mortality in Africa.^4^ PCV and rotavirus vaccines (RVs) were included in the WHO extended programme of immunisation (EPI) recommendations for routine vaccination following trial evidence that they prevent life-threatening infections.^5–7^

Reductions in pneumococcal morbidity and mortality following PCV introduction have been reported for high-income settings,^8–10^ alongside evidence of cost-effectiveness.^11 12^ Nicaragua, the first lower-middle income country to introduce PCV13 in 2010, demonstrated a 33% reduction in hospitalised pneumonia and inpatient case-fatality.^13^ Subsequent studies of PCV10 and PCV13 implementation from Peru, Brazil and Morocco have confirmed infant pneumonia mortality reductions, although with varied levels of impact (12% to 35%).^14–16^ Additionally, a modelled study from South Africa estimated a reduction of 61 pneumococcal-related child deaths per 100 000 child-years following PCV13 introduction.^17^

South Africa was the first African country to routinely introduce monovalent RV (RV1) in 2009, observing a 57% vaccine effectiveness (VE) against rotavirus hospitalisation, similar to the 64% VE reported from Malawi.^18 19^ Effectiveness against mortality has been estimated from middle-income settings,^20–22^ and Malawi has reported a 34% VE against vaccine age-eligible infant diarrhoeal mortality.^23^ However, population-level mortality impact of routine PCV (including 13-valent PCV (PCV13)) and sequential RV introduction in sub-Saharan Africa are yet to be reported.

Malawi, a low-income sub-Saharan African country, introduced PCV13 (Prevenar 13) in November 2011 into a PCV-naive population, using a 6, 10 and 14-week schedule and initial year-long three-dose catch-up for infants aged <12 months at introduction. RV1 (Rotarix) with a 6 and 10-week schedule, was introduced with no catch-up in October 2012. We aimed to prospectively evaluate population impact and effectiveness of PCV13, in the context of subsequent RV1 roll-out and on-going public health initiatives targeting child health, on non-traumatic mortality in vaccine age-eligible infants in Malawi.

## Methods

We conducted two prospective population birth cohort studies in the Northern (Study 1) and Central (Study 2) regions of Malawi (online supplementary eFigure 1).^24^ Study 1 investigated PCV13 impact, defined as the population-level reduction in infant mortality; Study 2 investigated both impact and effectiveness, with VE defined as the individual risk ratio in vaccinated versus unvaccinated infants.^25^

10.1136/bmjgh-2020-002669.supp1 Supplementary data

### Study 1

Study 1 was conducted at the Karonga Health and Demographic Surveillance Site (KHDSS). KHDSS was established in 2002, currently covering a population of 40 000 people in 41 villages, with one rural government hospital and four health centres.^26^ Demographic data are included from 1 January 2004 to 1 July 2019, and vaccine coverage data are complete up to the 1 July 2017. PCV13 was introduced on 12 November 2011, therefore all children born from 1 October 2011 onwards were 6 weeks old at introduction and eligible to receive dose-one. This study provides a pre–post impact evaluation of PCV13 introduction.

#### Data collection and cleaning

Births and deaths were recorded monthly by 230 community-based volunteers. Vaccine and socioeconomic status were collected for each household on an annual basis, using a rolling recensus by trained interviewers. Verbal autopsies (VAs) were conducted by medical assistants at a median of 1 month (range: 2 weeks to 20 months) following death, using a modified version of the WHO 2012 tool.^27^ All data underwent double entry into a Microsoft Access database and conflicts were flagged for cleaning.

#### Vaccine impact analysis

Impact in Study 1 was estimated ecologically using negative binomial regression of study area-wide annual trend in non-traumatic 14–51-week infant mortality pre-PCV13 and post-PCV13 introduction, adjusted for year to account for underlying downward trends in infant mortality and RV1 introduction. The annual trend was derived using locally weighted 12-month moving averaging as follows:

$${\hat{Y}}_{t}=\frac{1}{24}\left(Y_{t+6}+Y_{t-6}\right)+\frac{1}{12}\;\left(Y_{t}+Y_{t+1}+Y_{t-1}+Y_{t+2}+Y_{t-2}+Y_{t+3}+Y_{t-3}+Y_{t+4}+Y_{t-4}+Y_{t+5}+Y_{t-5}\right)$$

where ${\hat{Y}}_{t}$ and $Y_{t}$ are the trend estimate and the observed incidence at month *t*.

Additionally, we used a change-point model with the full time series to determine whether PCV13 and RV1 introduction occurred before significant trend changes in infant mortality. In change-point analysis an intervention time point is not prespecified, therefore, with fewer assumptions than interrupted time series analysis it assesses whether: (1) changes in incidence have occurred; (2) identifies the most likely time for the change point.^28^ We used the Stata—*bayesmh—*function to fit a negative binomial Bayesian model for the above specified locally weighted 12-month moving averaging annual trend. We used uninformative prior for the mean and a uniform prior for month (all values are equally likely), 50 000 MCMC iterations with 10 000 burn-in period and specified one change point. The resulting change point (month), pre and post change-point mean, mean ratio and the corresponding 95% credible intervals were calculated.

Postintroduction impact was ecologically estimated using negative binomial regression of study area-wide mortality versus monthly three-dose PCV13 population coverage, adjusted for year. Coverage was calculated as the cumulative number of infants who received three-doses of PCV13, divided by the cumulative total number of age-eligible infants residing in the study area and surviving to 14 weeks. Small population size precluded individual VE analysis.

### Study 2

Study 2 was conducted in Mchinji, a rural district with a population of 465 000 in 1832 villages, based on a census we conducted in March 2012. Healthcare was provided at 1 government hospital, 11 health centres, 354 community healthcare workers and 4 rural hospitals with limited inpatient facilities that provide care for a small fee. Cohort recruitment ran from 24 November 2011 (soon after PCV introduction) to 1 June 2015, and follow-up with mortality surveillance ran from 1 March 2012 (before RV introduction) to 1 June 2016. This site does not contribute any pre-PCV13 data, precluding a pre–post impact analysis.

#### Data collection

Pregnancies, pregnancy outcome and deaths in under 5 year olds were recorded monthly by 1059 volunteers. Field enumerators conducted household visits at 4 months and 1 year of age to collect vaccine status, socioeconomic variables and verify survivorship. Under-five deaths had a VA conducted at median 14 months (range: 2–50 months) following death using the WHO 2012 tool, with vaccine status recorded by senior monitoring and evaluation officers.^27^ Data were single entered into a Microsoft Access database with in-built validation rules and underwent automated monthly cleaning; errors in identification were sent for field verification. A random subset of 4-month and 1-year interviews were redone quarterly and all vaccine clinics were visited to audit documentation for quality control.

#### Sample size

The sample size for individual VE was calculated for 80% power to detect a 25% reduction in non-traumatic infant mortality, assuming 14–51-week infant mortality of 15/1000 live births, 80% three-dose vaccine coverage and 15% loss to follow-up. A sample of 45 520 births surviving to 14 weeks and 552 death events were required.

#### Vaccine impact analysis

In the absence of pre-PCV13 data, population-level impact in Study 2 was estimated using negative binomial regression of yearly mortality versus yearly vaccine coverage, by geographical cluster, adjusted for two-dose RV1 coverage. Coverage was calculated as the number of dose-eligible infants who received one, two or three doses of PCV13 by 52 weeks of age, divided by the total number of infants residing in the cluster and surviving to 14 weeks. Geographical clusters were 354 government-defined community healthcare worker catchment areas, with a median population of 1300 people (IQR: 984–1687).

#### PCV13 VE analysis

Unadjusted and adjusted individual-level VE of three versus zero dose PCV13 receipt against non-traumatic mortality in infants aged 14–51 weeks was estimated using Cox regression as the primary analysis.^29 30^ VE was derived as:

(1−HR)×100.

PCV13 doses received were modelled as time-dependent covariates, using date of vaccination recorded from caregiver-held health records (health passports) to split survival time into vaccinated and unvaccinated periods. Missing vaccination dates were imputed using chained equations with 10 imputations; all variables included in the primary model were used in the imputation (online supplementary eMethods 1). Infants who migrated did not contribute any survival time as vaccine status could not be determined.^31^ The proportional hazards assumption was tested using Schöenfeld’s residuals.

Decided a priori, analyses were adjusted for a range of potential confounding factors associated with both risk of mortality and vaccine uptake. These included maternal survival, education, age and marital status, household assets, household construction, water and sanitation facilities and a binary indicator of RV1 introduction. Individual RV1 and other EPI vaccine receipt (including *Haemophilus influenzae B* vaccine) were not included due to collinearity with PCV13 receipt, and too few children exclusively received PCV13 to conduct a subanalysis with this group. Distance to the nearest health facility (in kilometres) and season (rainy/dry) were investigated post hoc as possible proxies of vaccine access; however, neither showed any association with vaccine uptake, or survival, and were not included in the final model.

The following sensitivity analyses were conducted: Royston-Parmar flexible parametric survival models to describe time-varying vaccine effects^32^; using acute respiratory infection (including pneumonia), meningitis and sepsis-associated mortality and diarrhoea-related mortality as the outcome (with children who died of other causes excluded from the model); using individuals who survived to 6 and 26 weeks as the eligible population^30^; random effects models to account for cluster-level effects. Analyses were conducted using Stata SE V.14.

### Definitions

#### Exposure

We recorded receipt of zero, one, two and three doses of PCV13, with three versus zero doses as the primary exposure of interest. Vaccine date and receipt were collected during interviews and VAs from health passports, or caregiver recall if a written record was unavailable (online supplementary eMethods 2).

#### Primary outcome

We recorded deaths among three-dose eligible infants (ie, aged between 14 and 51 completed weeks) from a non-traumatic cause, as defined by the WHO 2012 VA guidelines (online supplementary eMethods 3). This primary outcome, although aetiologically non-specific has the advantage of being free from limitations in cause of death classification using VAs.^33^ Cause-specific mortality was included as a sensitivity analysis, using InterVA-4, a probabilistic Bayesian algorithm, to automate the analysis of the VAs and assign probability weighted cause of death.^34^ Overall, InterVA has been found to have reasonable agreement (concordance coefficient=0.81) with physician coded cause of death in infants, but was lower for acute respiratory infections in similar settings.^35^

### Patient and public involvement

Prior to the start of the study, the protocol was presented to the District Executive Committee and District Health Management teams in Mchinji and Karonga districts for input and approval. Extensive community engagement was conducted for this new data collection activity in Mchinji district, including the recruitment of village-level volunteers and meetings with traditional leaders and area development committees. Community consent was sought during study introduction.

### Ethics

Verbal informed consent was obtained for all interviews. The study was approved by the National Health Sciences Research Ethics Committee in Malawi [#837], London School of Hygiene and Tropical Medicine [#6047] and Centres for Disease Control and Prevention [#6268].

## Results

### Study 1

Prior to PCV13 introduction, between 1 January 2004 and 30 September 2011, 10 593 live births were recorded, with 9759 confirmed survivors at 1 year and 171 non-traumatic infant deaths between 14 and 51 completed weeks of age (mortality of 17/1000 live births). Post-PCV13 introduction, between 1 October 2011 and 1 July 2019, 9698 live births were recorded, with 8780 confirmed survivors at 1 year and 45 non-traumatic deaths between 14 and 51 completed weeks of age (mortality of 5/1000 live births, online supplementary eFigure 2.1). Median age at death was 31 weeks.

Health passports were seen in 67% of deceased and 92% of surviving infants. Postintroduction, overall three-dose PCV13 coverage was 46% and 94%, and two-dose RV1 coverage was 65% and 92% in age-eligible deceased and surviving infants, respectively. PCV13 doses were administered at a median of 8 (IQR: 7–10), 14 (IQR: 12–17) and 19 (IQR: 16–24) weeks.

**Figure 1:**
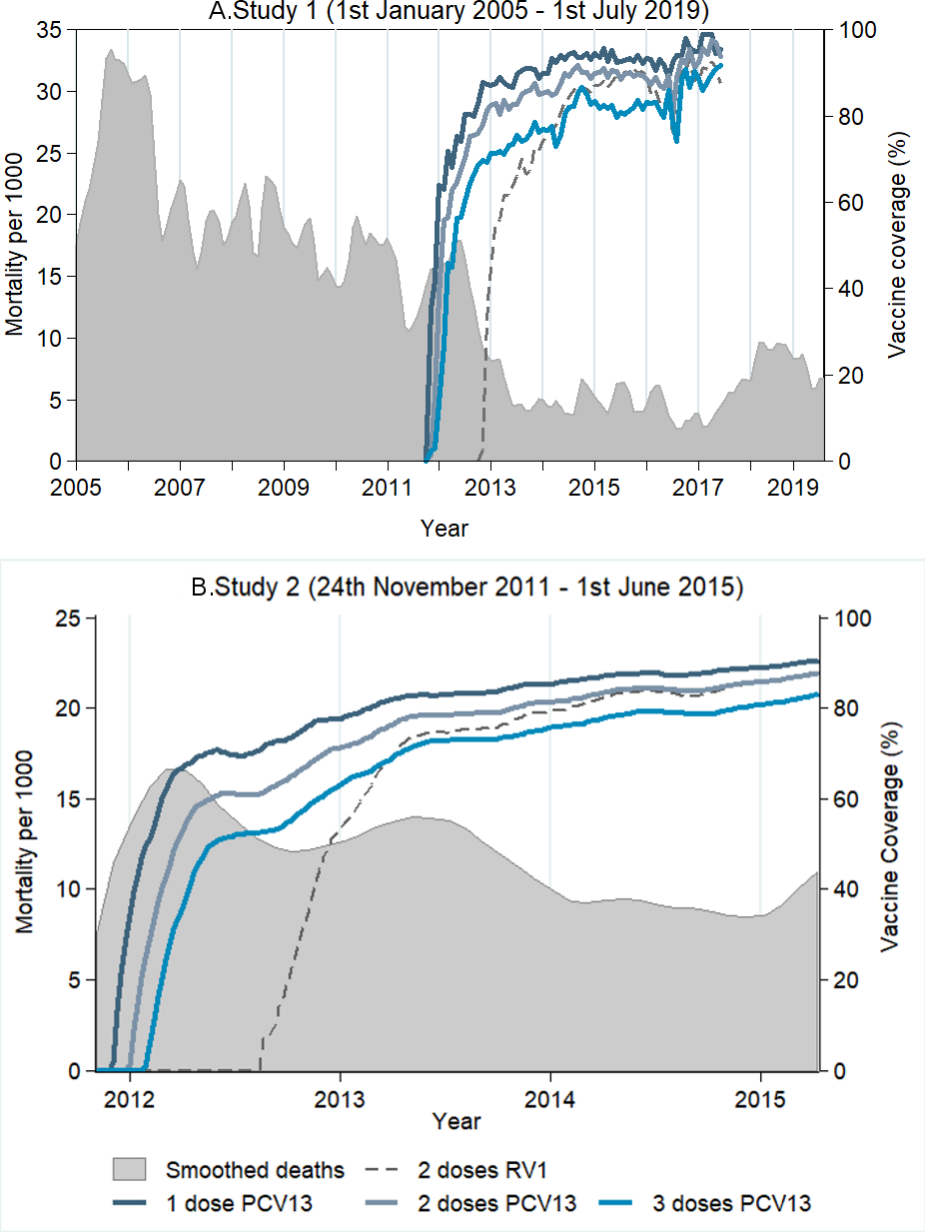
PCV13 and RV1 vaccine coverage and age-eligible infant deaths during the cohort period. (A) Study 1; (B) Study 2. Smoother in panel A: Smoother in panel B was done using locally weighted regression function, with a 0.15 bandwidth, due to the smaller number of time points. PCV, pneumococcal conjugate vaccine; RV, rotavirus vaccine.

Smoothed annual incidence data showed evidence of overdispersion. Pre–post negative binomial regression, adjusted for year, demonstrated a 28.6% (95% CI: 15.3 to 39.8; p value<0.001) reduction in non-traumatic infant mortality among age-eligible infants (figure 1A). A single change point in the trend of infant mortality was identified in November 2012 (credible Bayesian interval: June 2012 to June 2013). The pre change-point mean mortality was 19.04 (95% CI: 15.46 to 23.80), compared with a post change-point mean of 5.24 (95% CI: 4.12 to 6.73); the ratio of pre to post change-point mean was 3.69 (95% CI: 2.64 to 4.97; online supplementary eFigure 2.2).

**Figure 2:**
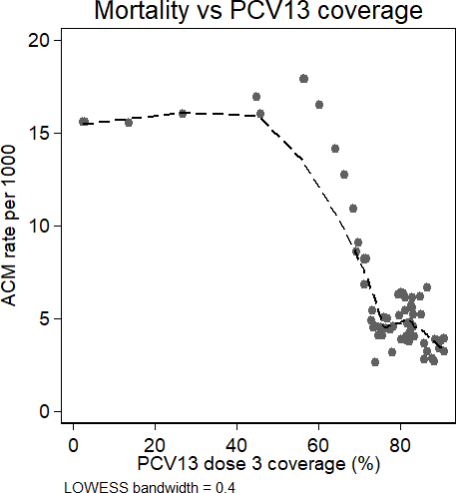
Smoothed trend in ACM against three-dose PCV13 coverage from Study 1. LOWESS smoothing bandwidth = 0.4. ACM, all-cause non-traumatic infant mortality; PCV, pneumococcal conjugate vaccine; LOWESS, Locally weighted smoothing.

Adjusted negative binomial regression of monthly mortality against three-dose coverage found every 1 percentage point increase in coverage was associated with a 2.0% (95% CI: 1.5 to 2.4; p value<0.001) decrease in mortality (figure 2).

### Study 2

**Figure 3:**
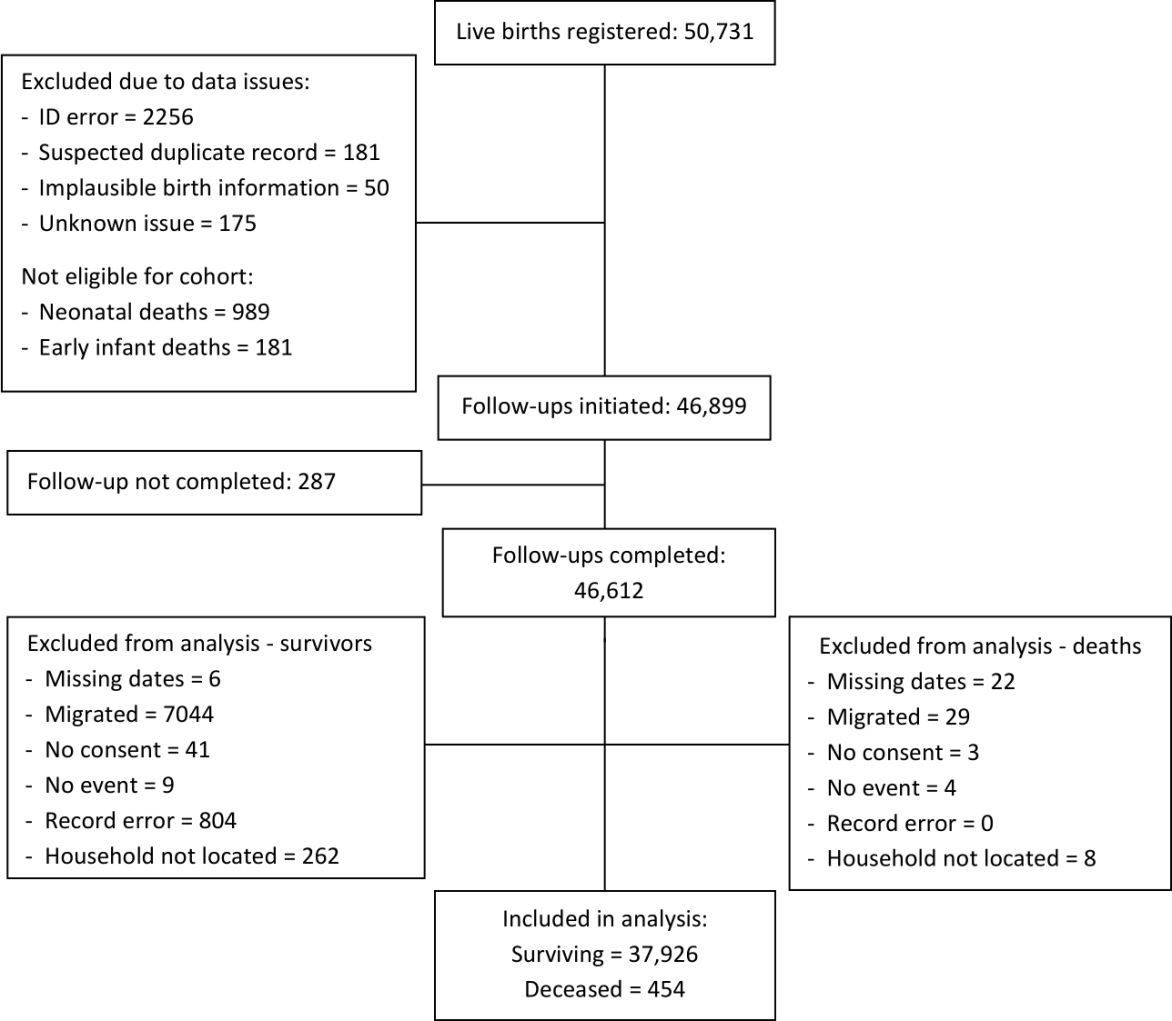
Cohort recruitment flow diagram for Study 2.

Between 24 November 2011 and 1 June 2015, we registered 50 731 live births; 454 infants died between 14 and 51 weeks of age and 37 926 were confirmed survivors at 1 year (figure 3). The crude birth rate was 32/1000 population and median age at death was 32 weeks. During the same period, stillbirth rates (23 and 24/1000 births in 2013 and 2015, respectively), and neonatal mortality rates (26 and 27/1000 live births in 2013 and 2015) remained stable. Additional descriptive parameters are presented in online supplementary eTable 1.1.

Health passports were seen in 89% of infants, with higher availability in surviving than deceased infants (90% vs 26%)—there was no difference in PCV13 coverage recorded between those with and without health passports in deceased infants (online supplementary eTable 1.2). A date of vaccination was available for 89% of children, with PCV13 doses administered at a median of 8 (IQR: 6–10), 14 (IQR: 11–16) and 20 (IQR: 16–22) weeks. Three-dose PCV13 coverage was 89% in surviving and 73% in deceased children (table 1); overall two-dose RV1 coverage was 73%. Following RV1 introduction 15 (<0.1%) children received RV1 but no PCV13, and 757 (3%) children received PCV13 without RV1. Between years 1 and 3 post-PCV introduction, non-traumatic age-eligible infant mortality decreased from 17 to 10/1000 live births (figure 1B).

**Table 1:** Description of Study 2 cohort, including vaccine and socioeconomic status by survival

Variable	Survived N (%)	Deceased N (%)
Total	37 926	454
PCV13 doses*		
0 doses	1657 (4)	43 (9)
1 dose	343 (1)	21 (5)
2 doses	1242 (3)	53 (12)
3 doses	33 703 (89)	331 (73)
Missing	982 (3)	6 (1)
RV1 introduction*		
Pre-RV1	9208 (24)	131 (29)
Post-RV1	28 718 (76)	323 (71)
Mother’s marital status*		
Married	34 055 (90)	345 (76)
Single	2023 (5)	46 (10)
Separated/widow	1752 (5)	47 (10)
Mother deceased	28 (0)	10 (2)
Missing	68 (0)	6 (1)
Mother’s education *		
None	4457 (12)	69 (15)
Primary	28 765 (76)	339 (75)
Secondary/tertiary	4631 (12)	39 (9)
Missing	73 (0)	7 (1)
House quality* †		
Worst	28 920 (76)	365 (80)
Middle	5637 (15)	51 (11)
Best	3305 (9)	32 (7)
Missing	64 (0)	6 (1)
Water source*		
Open source	7399 (20)	110 (24)
Protected source	30 476 (80)	338 (75)
Missing	51 (0)	6 (1)
Toilet facility*		
None	7334 (19)	73 (16)
Some	30 539 (81)	375 (83)
Missing	53 (0)	6 (1)

	Median (IQR)	Median (IQR)
Household assets*‡	1 (0–3)	1 (0–2)
Mother’s age at child’s birth*§	25 (21–31)	26 (22–33)

*P value<0.05 from χ^2^ test in categorical variables and t-test comparison of means in continuous variables between surviving and deceased children.

†House quality is a composite of the construction materials used to make the roof, walls and floor.

‡Household assets include: bicycle, radio, ox cart and mobile phone; mean in survived=1.5, mean in deceased=1.2.

§Mean in survived=26.1; mean in deceased=28.0.

PCV, pneumococcal conjugate vaccine; RV, rotavirus vaccine.

Across 354 geographical clusters, three-dose PCV13 coverage ranged from 58% to 100% and mortality ranged from 0 to 87/1000 live births. Cluster-level impact analysis, using negative binomial regression adjusted for RV1 coverage, found that every 1% absolute increase in three-dose coverage by geographical cluster was associated with a 1.3% reduction in non-traumatic infant mortality (95% CI: 0.3% to 2.4%; p value=0.02).

**Figure 4:**
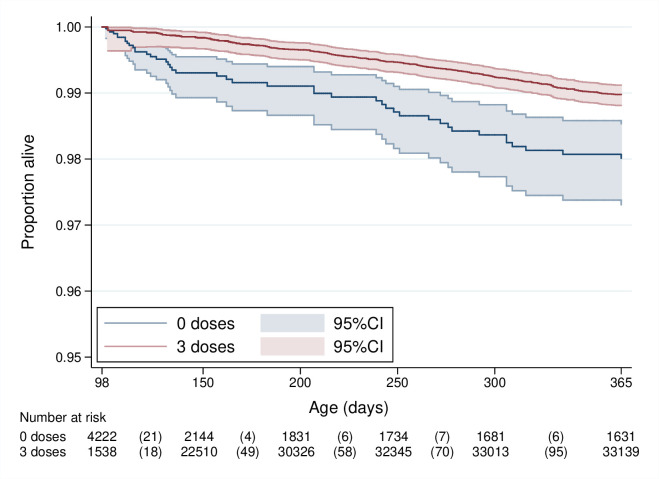
Kaplan-Meier survival curve (Study 2).

Adjusted Cox analysis, with time-dependent PCV13, estimated three-dose PCV13 VE to be 44.6% (95% CI: 23.0% to 59.1%) against non-traumatic age-eligible infant mortality (table 2, figure 4). Three-dose VE was 48.3% (95% CI: −5.9% to 74.1%) in InterVA-coded acute respiratory infection, meningitis and sepsis-associated mortality. Proportional hazards assumptions were not violated in either model (p value=0.63 and p value=0.10, respectively). When adjusted for RV1 introduction, three-dose PCV13 was not significantly associated with a reduction in diarrhoea-related mortality (VE: 18.5%; 95% CI: −57.7% to 57.8%).

**Table 2:** Adjusted Cox proportional hazards survival analysis of non-traumatic deaths in age-eligible infants (Study 2)

Variable	Adjusted HR*	95% CI	P value
PCV13 status			
0 doses	1.00		
1 dose	0.49	0.28 to 0.84	0.009
2 doses	0.65	0.44 to 0.96	0.029
3 doses	0.55	0.40 to 0.77	<0.001
RV1 introduction			
Pre-RV1	1.00		
Post-RV1	0.78	0.63 to 0.97	0.023
House†			
Worst	1.00		
Medium	0.74	0.54 to 1.02	0.063
Best	1.00	0.68 to 1.47	0.992
Mother’s marital status			
Married	1.00		
Single	2.33	1.67 to 3.23	<0.001
Separated/widowed	2.30	1.67 to 3.17	<0.001
Mother deceased	40.22	20.42 to 79.21	<0.001
Mother’s education			
None	1.00		
Primary	1.01	0.76 to 1.33	0.964
Secondary/tertiary	0.73	0.47 to 1.13	0.157
Water			
Protected source	1.00		
Open source	1.24	0.99 to 1.55	0.061
Toilet			
None	1.00		
Some facility	1.42	1.09 to 1.83	0.008
Household assets‡	0.82	0.74 to 0.90	<0.001
Mother’s age at birth	1.04	1.03 to 1.06	<0.001

Model satisfied proportional hazards assumption across all 10 imputations (average p value=0.629).

*All variables were included in the adjusted model.

†House quality is a composite of the construction materials used to make the roof, walls and floor.

‡Household assets include bicycle, radio, ox cart and mobile phone.

PCV, pneumococcal conjugate; RV, rotavirus vaccine.

Sensitivity analyses examined mortality by vaccine receipt from 6 weeks (one-dose eligibility) and 26-week survival (allowing for late dose-three receipt), giving a VE of 54.5% (95% CI: 48.8% to 66.1%) and 27.0% (95% CI: −15.8% to 54.0%), respectively. Proportional hazards were violated in the 6-week model but not the 26-week model (p value=0.003 and p value=0.58, respectively). Adjusting for individual and cluster-level random effects did not materially change the VE estimates, and these models were not statistically supported (online supplementary eTable 2.1). Royston-Parmar analysis, allowing for time-varying vaccine effects, demonstrated that the hazard rate varied over survival time (online supplementary eFigure 3).

## Discussion

In two prospective population-based, geographically independent cohort studies in Malawi, declines in vaccine age-eligible infant mortality were observed following the introduction of PCV13 and then RV1 into the Malawi EPI programme (figure 1). In both settings, these declines in infant mortality were associated with three-dose PCV13 coverage. In Northern Malawi, the effect has been sustained for nearly 8 years postintroduction. A change-point analysis identified a break point in the mortality trend in November 2012, a year after PCV13 introduction and shortly after RV1 introduction, supporting a vaccine-associated impact.

Measuring the effect of a new vaccine at the population level can be challenging, particularly in resource-limited settings and where background child mortality is changing rapidly.^36^ We studied two populations using different methods at scale, allowing for the measurement of plausible change and validation of our findings between studies. The communities participating in these studies were typical of many in rural Africa, with well-functioning EPI programmes but a high prevalence of undernutrition, HIV, malaria and resource-limited health systems. Our study sites had comparable birth and under 5-year-olds mortality rates to reported Malawian national averages pre-PCV13,^37^ suggesting our birth and death ascertainment had adequate population coverage. These two settings and their consistent results support the generalisability of our findings to other contexts in rural sub-Saharan Africa.

The VE estimate we observed was considerably higher than the 25% we anticipated and may reflect the true impact. It is plausible that a synergistic benefit from RV1 and PCV13 occurred that we could not account for in analysis. We were unable to directly adjust for individual-level RV1 receipt in this study, as children rarely received one vaccine but not the other, and therefore were not able to completely disentangle the effects.

It has been shown that pneumococcal pneumonia increases a child’s vulnerability to other infectious diseases,^38 39^ and that pneumonia often has multiple causative pathogens.^40 41^ Thus, reducing the incidence of pneumococcal infections likely impacts morbidity and mortality from common infections, such as malaria or viral respiratory infections, especially in the context of widespread undernutrition or anaemia. Indeed, our sensitivity analysis found only a slightly higher VE against suspected acute respiratory infections, meningitis and sepsis, than against all-cause mortality. Although, it is important to note that the delay between deaths and VA may have resulted in recall bias for specific symptoms, such as cough or difficulty breathing. In this context, planned evaluation of combined or sequential introduction of childhood vaccinations needs to carefully consider whether evidence of individual vaccine impact and effectiveness can be ascertained. Further programmatic investigation of the potential for additive benefits of these vaccines, in the context of on-going public health initiatives to reduce under 5-year-olds mortality is needed to optimise scale-up.

The impact estimate based on the pre–post analysis, accounting for background trends in infant mortality and RV1 introduction, is consistent with the finding from the 9-valent PCV trial in The Gambia,^42^ and RV1 introduction in low-income and middle-income countries.^20–22^ High vaccine coverage was rapidly achieved and prevaccine data have indicated the importance of infant mortality from respiratory and diarrhoeal disease in Malawi.^1 36 43^ A reduction in vaccine-type invasive pneumococcal disease following PCV13 introduction in Malawi has been reported from southern Malawi.^44 45^ Additionally, a concurrent study in central Malawi observed a 36% reduction in inpatient pneumonia mortality, and a 47% reduction in hypoxemic pneumonia.^45^ These simultaneous reductions in more specific pneumococcal and clinical endpoints add support to the finding that PCV13 was associated with a reduction in population-level non-traumatic infant mortality. A 64% reduction in hospitalised rotavirus diarrhoea has also been reported following RV1 introduction in Malawi.^19^ Thus given what is known about the individual effectiveness of these vaccines on disease-specific endpoints, an interaction between them would not have to be large to deliver major synergistic public health benefits.

Our data suggest that three-dose PCV13 receipt was more effective in younger infants. The sensitivity analysis of infants surviving to 26 weeks, by which time the majority of children had received all three-doses, found a lower and non-significant VE. The Royston-Parmar models, allowing VE to vary over survival time, supports the finding of differing effectiveness by age.^32^ However, this may be explained by epidemiological factors (eg, survivorship bias). Survivorship bias may also contribute to the apparent lack of a dose-response which we observed, as those surviving to three-dose eligibility are more robust than those who only survived long enough for one or two doses. As pneumonia-related deaths were concentrated in younger infants, programmatic emphasis on vaccination timeliness may optimise VE,^7 46^ although a recent modelling exercise found minimal impacts of PCV delays on mortality.^47^

As a large and complex observational field study, there may be residual confounding biassing our VE estimate. Although we adjusted for key sociodemographic confounders, it is unlikely we captured them all. We were also unable to adjust for individual exposure to concurrent community-based public health interventions which were on-going in both sites. Systematic monitoring of health systems performance and key child health indicators was not feasible, but should be recommended for future studies. In Study 2, a mentorship programme for paediatric pneumonia diagnosis, management and the introduction of pulse oximetry was implemented.^48^ If parents of vaccinated infants were more likely to seek healthcare, then vaccinated infants may have also received improved pneumonia case management. This would both reduce mortality in the vaccinated and magnify any underlying frailty bias (ie, if unvaccinated infants do not seek improved care), increasing apparent VE. However, random effects models that accounted for unmeasured cluster-level confounding did not change the VE estimates and were not statistically supported (online supplementary eTable 2.1). Nevertheless, the argument that children unable to access vaccines also lack access to treatment interventions only strengthens the argument for vaccine introduction and maximisation of population coverage, particularly among the most vulnerable.

Under-ascertainment of unvaccinated survivors, vaccinated non-survivors or misclassifying surviving unvaccinated infants as vaccinated could all increase the VE. Incomplete follow-up was higher in surviving infants, and if these lost infants were more likely to be unvaccinated (eg, children of migrant farm labourers with limited access to care) it would increase apparent VE by reducing unvaccinated survival time in the analysis. We were unable to assess differences between those who did and did not complete follow-up, although crude birth, mortality and vaccination rates were comparable to national estimates.

We recorded more health passports in surviving than deceased infants. It is common practice in Malawi to bury health passports when a child dies, leading to lower documentation of vaccine status from these children. Reliability of caregiver recall is varied and may be subject to recall and social desirability bias,^49–51^ the direction of which is unknown in our population. Relying solely on health passports would also have introduced bias, as it may be associated with higher healthcare engagement and vaccination. We conducted several quality assurance activities around vaccine status to mitigate these biases where possible and have previously published our decision process for including caregiver recall data.^30^ We compared vaccine status in deceased infants between those with and without a health passport and found similar rates (online supplementary eTable 1.2), suggesting that these biases are unlikely to have substantially influenced our VE estimate.

The mortality impacts we observed are likely attributable to more than prevention of severe life-threatening pneumococcal infections and severe rotaviral diarrhoea, and should be interpreted in the context of an existing functioning EPI programme, concurrent public health interventions and longer-term downward trends in infant mortality. Nonetheless, our results show significant declines in infant mortality in Malawi following sequential PCV13 and RV1 introduction, with adjustment for background trends. Considering prior trial evidence of efficacy for these vaccines against infant mortality, and the sustained impact over time, these findings support the continued investment in introduction and scale-up of these vaccines that target pneumonia and diarrhoea, particularly in high child mortality settings.

## Data Availability

Data are available upon request. Anonymised data are available upon request to Professor Neil French (N.French@liverpool.ac.uk), for the purposes of research only, and subject to approval form the National Health Sciences Research Ethics Committee of Malawi.
