# Supplementary material for: Population impact and effectiveness of sequential 13-valent pneumococcal conjugate and monovalent rotavirus vaccine introduction on infant mortality: prospective birth cohort studies from Malawi
Source: BMJ Glob Health. 2020 Sep 9;5(9):e002669. doi: 10.1136/bmjgh-2020-002669 (PMC7482521; doi:10.1136/bmjgh-2020-002669)

Supplementary Materials:

eFigure 1: Map of study sites

eMethods 1: Multiple imputation description

eMethods 2: Vaccine status construction

eMethods 3: Definition use for cause of death

eFigures 2: Additional Study 1 figures

eTables 1: Additional Study 2 description tables

eTables 2: Sensitivity survival analysis and Royston-Parmer model for Study 2

eFigure 3: Royston-Parmar Model for Study 2

King C, *et al. BMJ Global Health* 2022; 5:e002669. doi: 10.1136/bmjgh-2020-002669

eFigure 1: Map of study sites

KHDSS: Karonga Prevention study demographic surveillance site

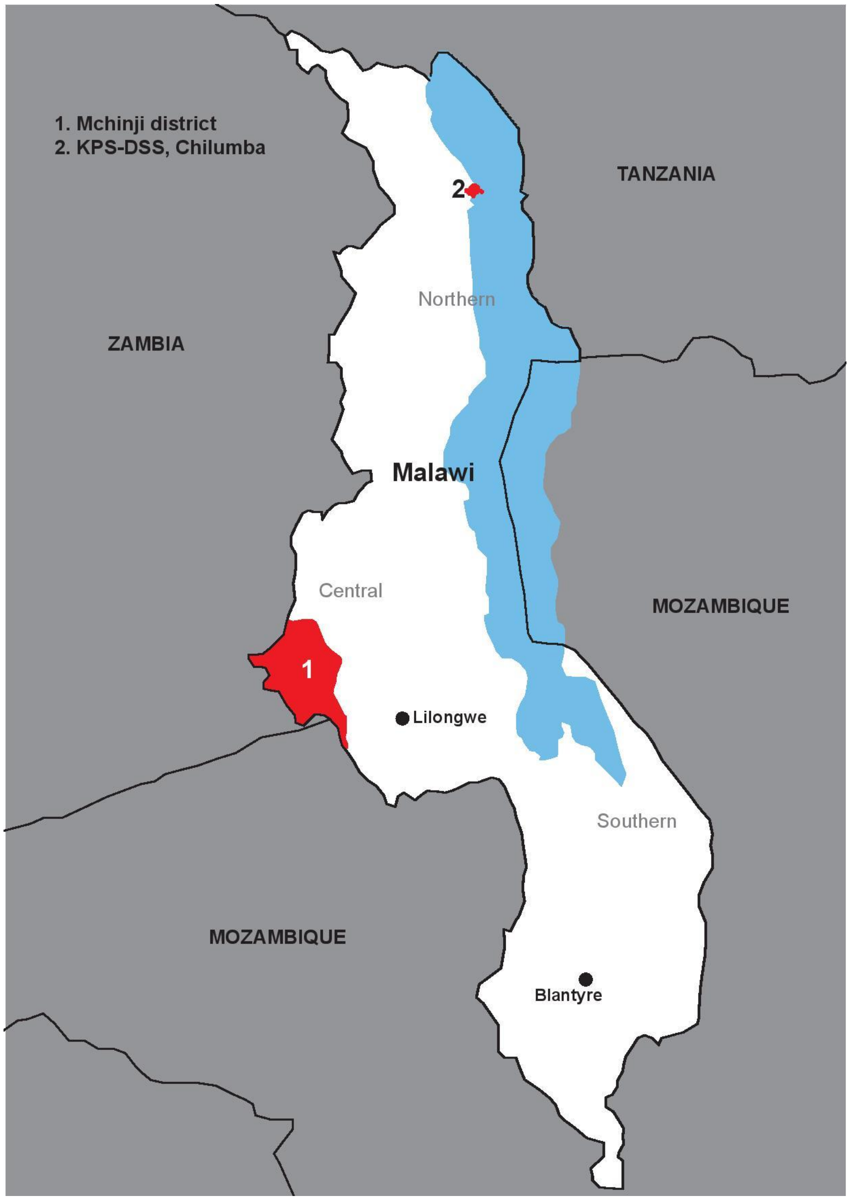

eMethods 1: Multiple imputation description

Multiple imputation using chained equations, 10 imputations were conducted with the following conditional models:

Mother's age at birth: truncreg motherage_birth age_pcv1 age_pcv2 age_pcv3 i.hhassets i.house toilet watersource i.motherhighestedu i.mothermaritalstatus survived , ll(14) ul(59)

Age of PCV1 receipt (days): truncreg age_pcv1 motherage_birth age_pcv2 age_pcv3 i.hhassets i.house toilet watersource i.motherhighestedu i.mothermaritalstatus survived , ll(1) ul(365)

Age of PCV2 receipt (days): truncreg age_pcv2 motherage_birth age_pcv1 age_pcv3 i.hhassets i.house toilet watersource i.motherhighestedu i.mothermaritalstatus survived , ll(1) ul(365)

Age of PCV3 receipt (days): truncreg age_pcv3 motherage_birth age_pcv1 age_pcv2 i.hhassets i.house toilet watersource i.motherhighestedu i.mothermaritalstatus survived , ll(1) ul(365)

Date of vaccination for each dose of PCV was then calculated as the date of birth plus the imputed age of dose receipt.

eMethods 2: Vaccine status construction

There are three sources of vaccine status information available for this cohort:

- Health passports (government issued caregiver-held documents)
- Caregiver recall
- Under 1 government vaccine registers (filled by healthcare workers at the point of vaccination and stored in frontline health facilities)

We ask to see health passports at routine interviews when children were 4 months and 1 year of age and at verbal autopsy interviews. In the absence of health passport, caregivers were asked to recall vaccine status. Reasoning for assigning levels of reliability to different sources is summarized below:

Data Source	Strengths	Weaknesses	Reliability
Health passport	• Filled in at the point of vaccination<br>• Dates included<br>• Less than 5% mis-recording	• Differential availability according to survival status	High
Under 1 register	• Routine data, therefore should be available for all, irrespective of survival status	• Some registers are missing or of very poor quality<br>• Tracing children through registers and across facilities is difficult<br>• Absence of record does not mean children are unvaccinated	Medium
Caregiver recall with known dates	• Dates included<br>• Generally some documented evidence provided e.g. twins health passport	• Uncommon	High
Caregiver recall of no vaccinations	• Generally anecdotal support which makes it believable	• Uncommon<br>• Relies on accurate recall	High
Caregiver recall	• Available for most children, regardless of survival status	• Recall bias and social-desirability bias (in both directions), so hard to adjust for the uncertainty<br>• Chance of interviewer bias	Low

Based on the strengths and weaknesses within each source of vaccine data, the following rules were applied to construct a binary variable indicating whether PCV13 was received or not received:

- If a vaccine is 'received' in the health passport at VA or 1-year interview, this information will be taken as correct and no modifications made to this vaccine
- If a vaccine is 'not received' or 'missing' at VA or 1-year interview, or no health passport was seen at these interviews:
  - If available, the vaccine status from a health passport at the 4-month interview will be used
  - If vaccines have been recorded in the under 1 register with evidence of a date of vaccination, this vaccine status will be used
  - If there is a conflict in data between data from the 4-month, the under 1 register or maternal report, information from the health passport will be taken as the correct, followed by the under 1 register and then maternal report.

eMethods 3: Definition used for cause of death

Accessed from (02/17): http://www.who.int/healthinfo/statistics/WHO_VA_2012_RC1_Instrument.pdf

WHO 2012 categorizations of cause of death given by InterVA to determine non-trauma infant deaths		
**VA code**	**ICD-10 code**	**Definition**
**Non-trauma**		
01.01	A41	Sepsis
01.02	J22, J18	Acute respiratory infection, including pneumonia
01.03	B24	HIV/AIDS related death
01.04	A09	Diarrhoeal diseases
01.05	B54	Malaria
01.06	B05	Measles
01.07	G03, G04	Meningitis an encephalitis
01.08	A35	Tetanus (excluding neonatal tetanus)
01.09	A16	Pulmonary tuberculosis
01.10	A37	Pertussis
01.11	A99	Haemorrhagic fever
01.99	B99	Other and unspecified infectious diseases
03.01	D64	Severe anemia
03.02	E46	Severe malnutrition
03.03	E14	Diabetes mellitus
04.01	I24	Acute cardiac disease
04.03	D57	Sickle cell with crisis
04.99	199	Other and unspecified cardiac disease
05.02	J45	Asthma
06.01	R10	Acute abdomen
07.01	N19	Renal failure
08.01	G40	Epilepsy
.98	R99	Other and unspecified non-communicable diseases
**Trauma**		
12.01	V89	Road traffic accident
12.02	V99	Other transport accident
12.03	W19	Accidental fall
12.04	W74	Accidental drowning and submersion
12.05	X09	Accidental exposure to smoke, fire and flames
12.06	X29	Contact with venomous animals and plants
12.07	X49	Accidental poisoning and exposure to noxious substance
12.09	Y09	Assault
12.10	X39	Exposure to force of nature
12.99	X59	Other and unexpected external cause of death
**Cause of death unknown**		
.99	R99	Cause of death unknown

King C, *et al. BMJ Global Health* 2022; 5:e002669. doi: 10.1136/bmjgh-2020-002669

eFigure 2: Additional Study 1 figures

eFigure 2.1: Study 1 participant inclusion

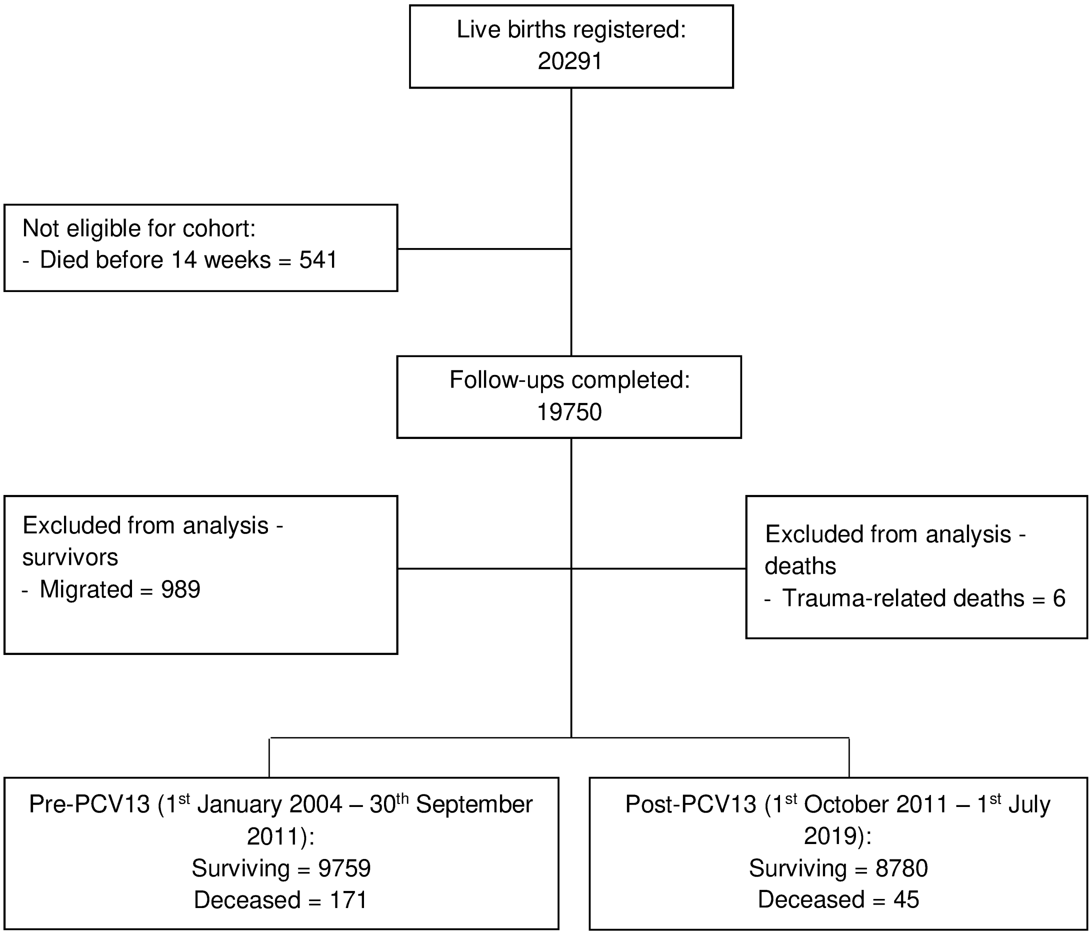

eFigure 2.2: Change-point analysis model diagnostics

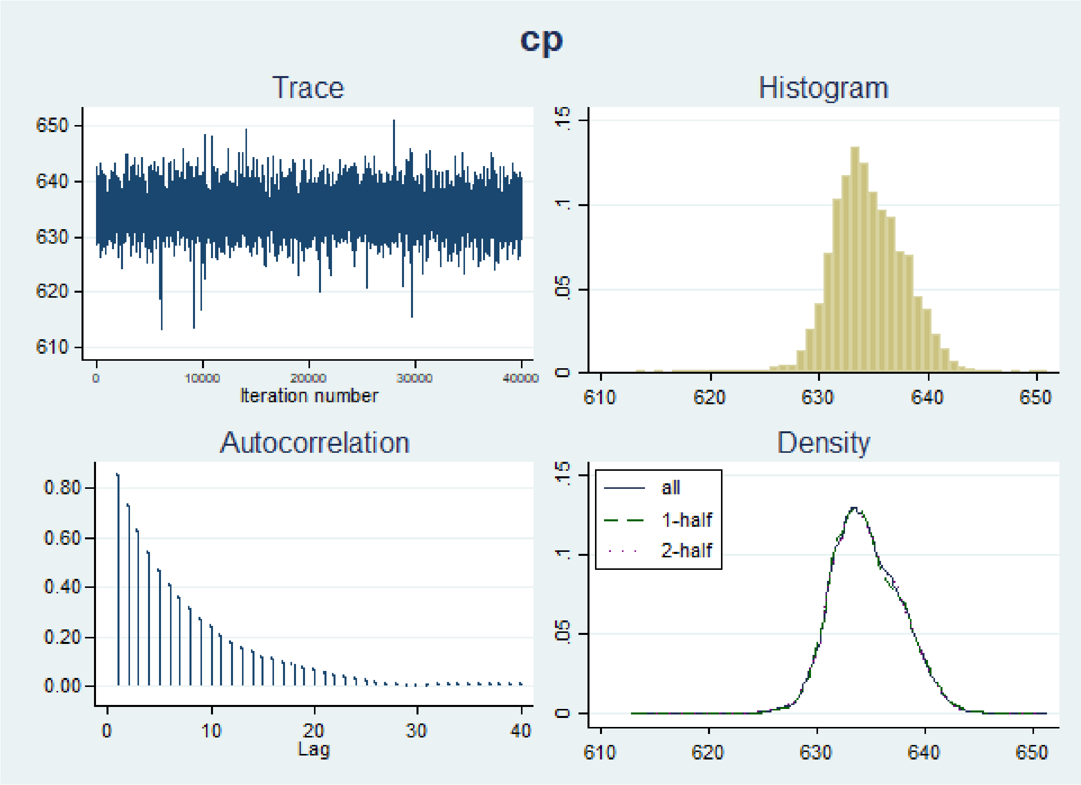

eTable 1: Additional cohort description in Study 2

eTable 1.1 – Socio-economic indicators during the cohort period in Study 2

	2012*	2013	2014	2015
Crude birth rate**	32	31	31	-
Under-5 mortality ***	55.2 / 1,000	59.0 / 1,000	47.9 / 1,000	46.9 / 1,000
Stillbirth	-	22.5 / 1,000	24.7 / 1,000	23.5 / 1,000
Post-neonatal infant mortality	17.1 / 1,000	17.6 / 1,000	13.2 / 1,000	15.9 / 1,000
3-dose PCV coverage	83%	87%	91%	91%
3-dose DPT-Penta coverage	90%	92%	94%	94%
2-dose RV1 coverage	32%	90%	94%	95%
Health passport available	86%	90%	92%	89%
Health facility delivery	92%	94%	94%	95%

*There was a delay in establishing the recording of stillbirth and early neonatal death outcomes in 2012, with this system fully established in October 2012 – therefore values for stillbirth have not been reported and under-5 rate may be slightly under reported.
**The total population of Mchinji was 465,000 based on a population census completed in March 2012. CBR for 2015 not calculated as we do not have a full year of birth data, and there are seasonal trends in births.
***Mortality rates are presented per 1,000 livebirths

eTable 1.2 – Vaccine status according to information source

PCV doses	Total		Survived		Deceased*	
	Reliable	Unreliable	Reliable	Unreliable	Reliable	Unreliable
0 doses	395 (1%)	1305 (31%)	384 (1%)	1273 (33%)	11 (8%)	32 (10%)
1 dose	274 (1%)	90 (2%)	266 (1%)	77 (2%)	8 (6%)	13 (4%)
2 doses	1046 (3%)	249 (6%)	1036 (3%)	206 (5%)	10 (8%)	43 (13%)
3 doses	32415 (95%)	1618 (38%)	32314 (95%)	1388 (35%)	101 (78%)	230 (72%)
Missing	9 (0%)	979 (23%)	9 (0%)	973 (25%)	-	3 (1%)

Reliable and unreliable are defined in eMethods 2
*There was no statistical difference in PCV13 doses received by source of vaccine data in deceased infants.

King C, *et al. BMJ Global Health* 2022; 5:e002669. doi: 10.1136/bmjgh-2020-002669

eTable 1.3 – Vaccine status and socio-economic associations

		**PCV13 – 0 doses Total =1,700**	**PCV13 – 3 doses Total = 34,033**
Mother's marital status*	Married	1,478 (87%)	30,671 (90%)
	Single	113 (7%)	1,783 (5%)
	Separated/widow	102 (6%)	1,525 (4%)
	Died	3 (0%)	28 (0%)
	Missing	4 (0%)	26 (0%)
Mother's education*	None	280 (16%)	3,817 (11%)
	Primary	1,288 (76%)	25,860 (76%)
	Secondary/tertiary	128 (8%)	4,321 (13%)
	Missing	4 (0%)	35 (0%)
House quality* ~	Worst	1,337 (79%)	25,877 (76%)
	Middle	255 (15%)	5,082 (15%)
	Best	104 (6%)	3,049 (9%)
	Missing	4 (0%)	25 (0%)
Water source*	Open source	496 (29%)	6,356 (19%)
	Protected source	1,200 (71%)	27,668 (81%)
	Missing	4 (0%)	14 (0%)
Toilet facility*	None	415 (24%)	6,370 (19%)
	Some	1,281 (75%)	27,646 (81%)
	Missing	4 (0%)	17 (0%)
		**Mean (SD)**	**Mean (SD)**
Household assets* #		1.3 (1.2)	1.5 (1.2)
Mother's age*†		27.8 (6.7)	27.1 (6.6)

* p-value<0.05 from Chi2 or t-test. ~ House quality is a composite of materials used for the roof, walls and floor. # Household assets include: bicycle, radio, ox cart and mobile. † Mother's age is standardized to be the age at birth.

eTable 2: Sensitivity survival analysis and Royston-Parmer model for Study 2

eTable 2.1: Random effects frailty model

Covariate		Hazard ratio	95% CI	p-value
PCV13 status	0 doses	1.00		
	1 dose	0.51	0.30, 0.88	0.015
	2 doses	**0.67**	**0.45, 0.98**	**0.039**
	3 doses	**0.53**	**0.38, 0.74**	**<0.001**
RV1 introduction	Pre-RV1	1.00		
	Post-RV1	**0.79**	**0.64, 0.98**	**0.031**
House	Worst	1.00		
	Medium	0.74	0.54, 1.01	0.060
	Best	1.01	0.68, 1.48	0.978
Marital status	Married	1.00		
	Single	**2.34**	**1.68, 3.25**	**<0.001**
	Separated/widowed	**2.32**	**1.68, 3.20**	**<0.001**
	Mother deceased	**41.95**	**20.97, 83.91**	**<0.001**
Mother's education	None	1.00		
	Primary	1.01	0.76, 1.33	0.953
	Secondary/tertiary	0.73	0.47, 1.13	0.162
Water	Protected source	1.00		
	Open source	1.23	0.98, 1.55	0.071
Toilet	None	1.00		
	Some facility	**1.46**	**1.12, 1.90**	**0.005**
Household Assets		**0.82**	**0.75, 0.90**	**<0.001**
Mother's age at birth		**1.04**	**1.03, 1.06**	**<0.001**

Gompertz survival distribution and Gamma frailty distribution.
Average Likelihood ratio test across 10 imputations: p-value = 0.093

eTable 2.2: Multi-level survival analysis

**Covariate**		**Hazard ratio**	**95% CI**	**p-value**
PCV13 status	0 doses	1.00		
	1 dose	**0.50**	**0.29, 0.87**	**0.013**
	2 doses	**0.66**	**0.45, 0.97**	**0.036**
	3 doses	**0.54**	**0.39, 0.74**	**<0.001**
RV1 introduction	Pre-RV1	1.00		
	Post-RV1	**0.79**	**0.64, 0.98**	**0.031**
House	Worst	1.00		
	Medium	0.73	0.53, 1.01	0.057
	Best	1.00	0.68, 1.47	0.996
Marital status	Married	1.00		
	Single	**2.35**	**1.69, 3.27**	**<0.001**
	Separated/widowed	**2.32**	**1.68, 3.20**	**<0.001**
	Mother deceased	**41.02**	**20.61, 81.66**	**<0.001**
Mother's education	None	1.00		
	Primary	1.02	0.77, 1.35	0.897
	Secondary/tertiary	0.74	0.48, 1.15	0.184
Water	Protected source	1.00		
	Open source	1.23	0.98, 1.55	0.073
Toilet	None	1.00		
	Some facility	**1.46**	**1.12, 1.90**	**0.005**
Household Assets		**0.82**	**0.75, 0.90**	**<0.001**
Mother's age at birth		**1.04**	**1.03, 1.06**	**<0.001**

Catchment area variance = 0.076 (95% CI: 0.01, 0.40)
Average Likelihood ratio test across 10 imputations: p-value = 0.098
Modelled using the Weibull distribution with two levels (level 1 = individuals; level 2 = community healthcare worker catchment area).
'mestreg' is not supported in multiply imputed data, the model presented here is from imputation 5 of 10 imputations using chained equations.
The 3-doses VE ranged from 46.0% - 46.6%

eTable 2.3: Cause-specific Cox model

**Covariate**		**Hazard ratio**	**95% CI**	**p-value**
PCV13 status	0 doses	1.00		
	1 dose	**3.55**	**2.04, 6.17**	**<0.001**
	2 doses	**2.41**	**1.42, 4.09**	**0.001**
	3 doses	0.62	0.36, 1.06	0.080
RV1 introduction	Pre-RV1	1.00		
	Post-RV1	**0.74**	**0.58, 0.94**	**0.014**
House	Worst	1.00		
	Medium	0.83	0.58, 1.18	0.302
	Best	1.29	0.86, 1.94	0.212
Marital status	Married	1.00		
	Single	**2.59**	**1.77, 3.79**	**<0.001**
	Separated/widowed	**1.75**	**1.14, 2.67**	**0.010**
	Mother deceased	**-**	**-**	**-**
Mother's education	None	1.00		
	Primary	**1.70**	**1.17, 2.49**	**0.006**
	Secondary/tertiary	1.29	0.75, 2.22	0.351
Water	Protected source	1.00		
	Open source	0.97	0.73, 1.28	0.828
Toilet	None	1.00		
	Some facility	**1.59**	**1.15, 2.19**	**0.005**
Household Assets		**0.88**	**0.79, 0.98**	**0.023**
Mother's age at birth		**1.06**	**1.04, 1.08**	**<0.001**

Average test of proportional hazards across 10 imputations: p-value = 0.101

eTable 2.4 Different survival cut-offs for vaccine effectiveness (Study 2)

		6-week survival			26-week survival		
**Covariate**		**HR**	**95% CI**	**p-value**	**HR**	**95% CI**	**p-value**
PCV13 status	0 doses	1.00			1.00		
	1 dose	**0.52**	**0.37, 0.73**	**<0.001**	0.98	0.39, 2.44	0.961
	2 doses	**0.48**	**0.34, 0.67**	**<0.001**	0.89	0.48, 1.65	0.716
	3 doses	**0.46**	**0.34, 0.61**	**<0.001**	0.73	0.46, 1.16	0.181
RV1 introduction	Pre-RV1	1.00			1.00		
	Post-RV1	**0.74**	**0.61, 0.91**	**0.004**	**0.72**	**0.56, 0.93**	**0.012**
House	Worst	1.00			1.00		
	Medium	0.89	0.68, 1.17	0.416	0.77	0.53, 1.13	0.186
	Best	0.94	0.65, 1.36	0.743	0.92	0.57, 1.51	0.749
Mother’s marital status	Married	1.00			1.00		
	Single	**2.07**	**1.53, 2.81**	**<0.001**	**2.20**	**1.46, 3.33**	**<0.001**
	Separated/widowed	**2.13**	**1.58, 2.86**	**<0.001**	**2.51**	**1.72, 3.65**	**<0.001**
	Mother deceased	**56.25**	**32.73, 96.68**	**<0.001**	**41.90**	**18.28, 96.06**	**<0.001**
Mother’s education	None	1.00			1.00		
	Primary	1.03	0.79, 1.33	0.835	1.02	0.73, 1.43	0.922
	Secondary/tertiary	0.81	0.54, 1.20	0.288	0.68	0.39, 1.18	0.171
Water	Protected source	1.00			1.00		
	Open source	1.20	0.98, 1.47	0.080	1.06	0.80, 1.40	0.704
Toilet	None	1.00			1.00		
	Some facility	**1.33**	**1.05, 1.67**	**0.016**	**1.44**	**1.05, 1.98**	**0.023**
Household Assets		**0.81**	**0.74, 0.88**	**<0.001**	**0.80**	**0.72, 0.90**	**<0.001**
Mother’s age at birth		**1.03**	**1.02, 1.05**	**<0.001**	**1.04**	**1.03, 1.06**	**<0.001**

Average test of proportional hazards across 10 imputations (6-week): p-value = 0.003
Average test of proportional hazards across 10 imputations (26 week): p-value = 0.581

King C, *et al. BMJ Global Health* 2022; 5:e002669. doi: 10.1136/bmjgh-2020-002669

eFigure 3: Royston-Parmar Model

Allowing the vaccine effectiveness to change over survival time (in this case the same as age), demonstrated that VE was higher in younger infants and after 6-months of age VE trended to no effect.

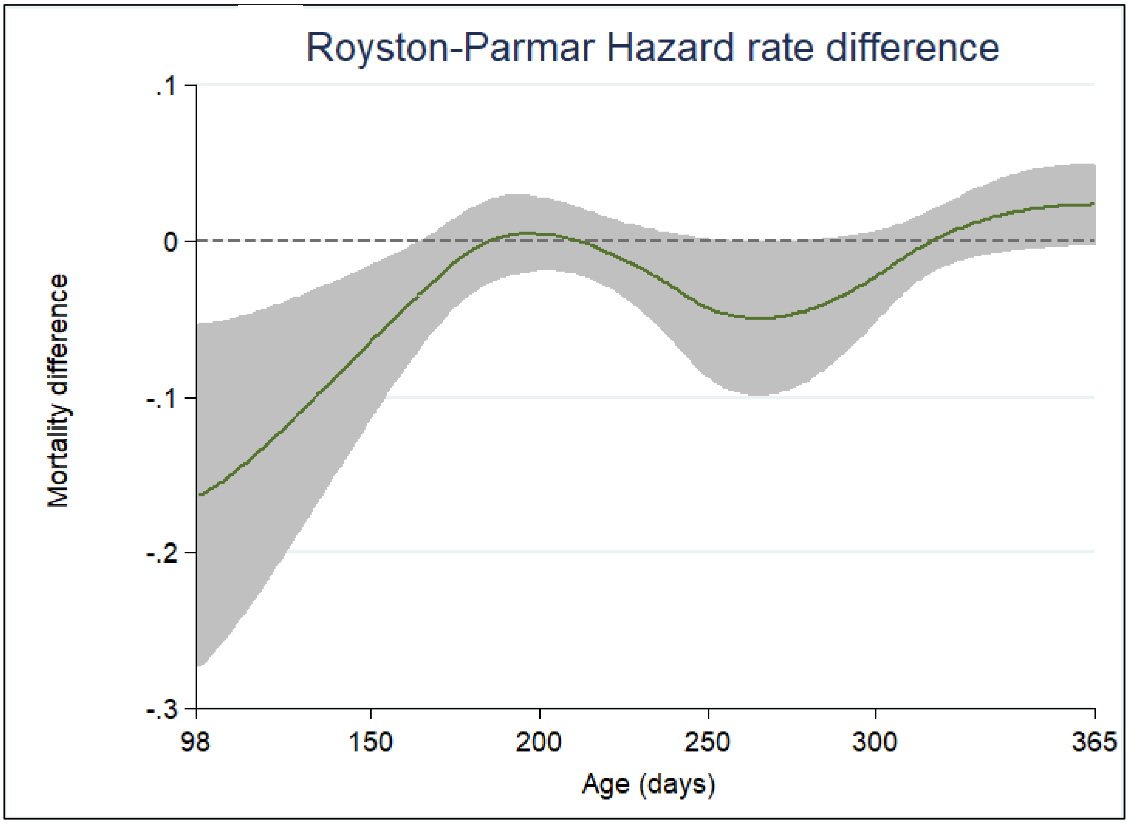

Supplement: Supplementary data [file bmjgh-2020-002669supp001.pdf]
